# A Novel Dynamic Compression Angle-Stable Interlocking Intramedullary Nail: Description, Validation, and Model Evaluation

**DOI:** 10.1155/vmi/7875699

**Published:** 2025-04-10

**Authors:** Luís Gustavo Gosuen Gonçalves Dias, Thiago André Salvitti Sá Rocha, Caio Afonso Santos Malta, Bruno Watanabe Minto, Alefe Luiz Caliani Carrera

**Affiliations:** ^1^Department of Veterinary Clinics and Surgery, School of Agricultural and Veterinarian Sciences of the São Paulo State University (FCAV UNESP), Jaboticabal, State of São Paulo, Brazil; ^2^Department of Veterinary Medicine, Federal University of Jatai (UFJ), Jataí, State of Goiás, Brazil

**Keywords:** biological osteosynthesis, experimental implants, implants engineer, intramedullary fracture fixation, long bone fractures

## Abstract

The stabilization of long-bone fractures using intramedullary nails offers significant biological advantages for bone healing. Nevertheless, the mechanical stability of the implant–bone interface remains suboptimal due to the absence of models capable of generating interfragmentary compression at the fracture site. To address these limitations, this study aims to describe and evaluate a novel dynamic compression angle-stable interlocking intramedullary nail (DCASIN), designed for use in conjunction with a compression device (CD). Its performance was compared with conventional and angle-stable interlocking intramedullary nails. Implantation was demonstrated using a tube-based bone model with transverse fractures. Compression was achieved in the proximal aspect of the DCASIN through an oblong hole that allowed the insertion of a Steinmann pin, which was then subjected to the thrust of the CD's primary screw (PS). To evaluate dynamic compression, a load cell connected to the Arduino/Genuíno Uno software was utilized. Three groups of interlocking nails were assessed: G1 (conventional), G2 (angle-stable), and G3 (DCASIN), with measurements taken at four time points (M1: prelocking, M2: after the first screw or PS for the DCASIN, M3: after the second implant, and M4: one-minute post-M3). No statistically significant differences in compression forces were observed for G1 and G2 across the measured time points. In contrast, G3 exhibited significantly higher compression at M2 than at M3 and M4, and its compression forces at M2, M3, and M4 were significantly greater than those in G1 and G2. Finite element analysis revealed no significant deformation in G3 during compression. In conclusion, the DCASIN combined with the CD achieved and sustained superior compression forces compared to conventional and angle-stable nails, thereby offering a promising alternative for the internal fixation of long bones.

## 1. Introduction

Transverse fractures of long bones are suitable for rigid osteosynthesis stabilization and interfragmentary compression, facilitating primary bone healing [1, 2], which is routinely achieved using dynamic compression plates [3]. However, the periosteal vascular tissue is essential for the bone healing process, and these plates are associated with damage to the vessels. This is a significant drawback in the decision-making process, along with the requirement for extensive soft tissue exposure during the surgical approach [1, 4].

Otherwise, angle-stable interlocking intramedullary nails (ASINs) demonstrated better biological maintenance during osteosynthesis due to the ability to perform the surgical procedure using minimally invasive osteosynthesis or the “open but do not touch” approach. This technique ensures the preservation of the primary clot and periosteal vascularization tissue [5–7]. However, commercially available interlocking nails are designed only for relative stabilization of fractures, characterized by an extensive working length, which results in a secondary bone healing process. Consequently, their use is restricted to fractures that require treatment through indirect ossification [8–10].

The time required for complete bone healing for transverse fractures that have undergone stabilization by dynamic compression is shorter than the other that have not undergone compression, the weight bearing is premature, and the complication rates are reduced [11]. This way, adequate dynamic compression for transverse fractures is desirable to obtain better outcomes, which encouraged the design of interlocking intramedullary nails that were able to promote interfragmentary compression during osteosynthesis [11, 12]. However, the previous devices had the disadvantage of not having angle-stable, which could lead to unstable fixation and malunion [13, 14].

In the given context, the necessity of designing an ASIN with dynamic compression is underscored, considering that a similar model has not yet been introduced in veterinary medicine. The authors hypothesize that the creation of such a device holds significance and efficacy for biological approaches and minimally invasive osteosynthesis, particularly in the treatment of transverse fractures. Additionally, it is suggested that this development could pave the way for similar models to be utilized in osteosynthesis for humans with similar objectives [15]. Moreover, the objective of this study was to delineate the development and functional pathways of a novel model of dynamic compression angle-stable interlocking intramedullary nail (DCASIN), along with comparative assessments of compression forces vis-à-vis conventional and angle-stable interlocking nails that were previously available.

## 2. Materials and Methods

The research received approval from the Ethics Committee for Animal Use at the School of Agricultural and Veterinary Studies, São Paulo State University, under protocol 012836/19. Additionally, the compression device (CD) developed in this study was patented by the Brazilian National Institute of Industrial Property (BR 10 2018 016021 4). The research comprised two phases: firstly, the design of the DCASIN and the establishment of its functional pathways for interfragmentary compression; secondly, the objective measurement of compression and comparative analysis against conventional interlocking intramedullary nail (CIN) and ASIN. All the graphical illustrations presented in this section were designed in the SolidWorks software (Dassault Systèmes, SolidWorks Corporation, Waltham, MA, USA).

### 2.1. Design and Development of the DCASIN and CD

**Figure 1:**
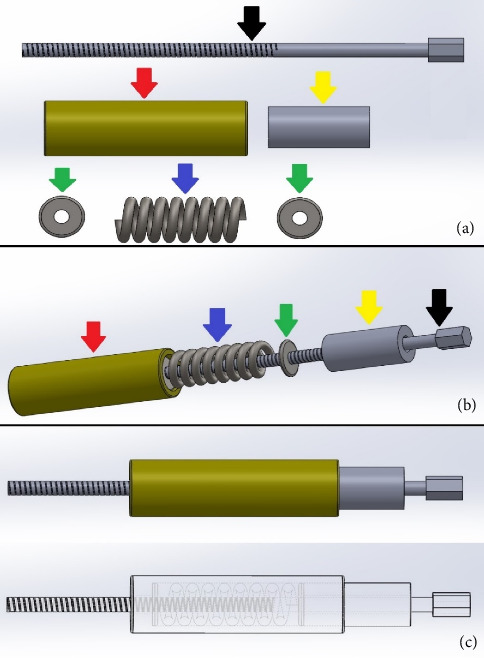
Prototype illustration of the components of the compression device (CD) designed for dynamic compression when applied with the dynamic compression angle-stable interlocking intramedullary nail (DCASIN). (a) Components of the CD, with the primary screw (PS) (black arrow), spring retainer (red arrow), compression measurer (yellow arrow), washers (green arrow), and spring (blue arrow). (b) Sequential insertion of the components of the CD, starting with the compression measurer inserted into the PS, followed by the spring retainer housing a spring held by two washers. (c) Final apparatus and transparency vision of the CD.

**Figure 2:**
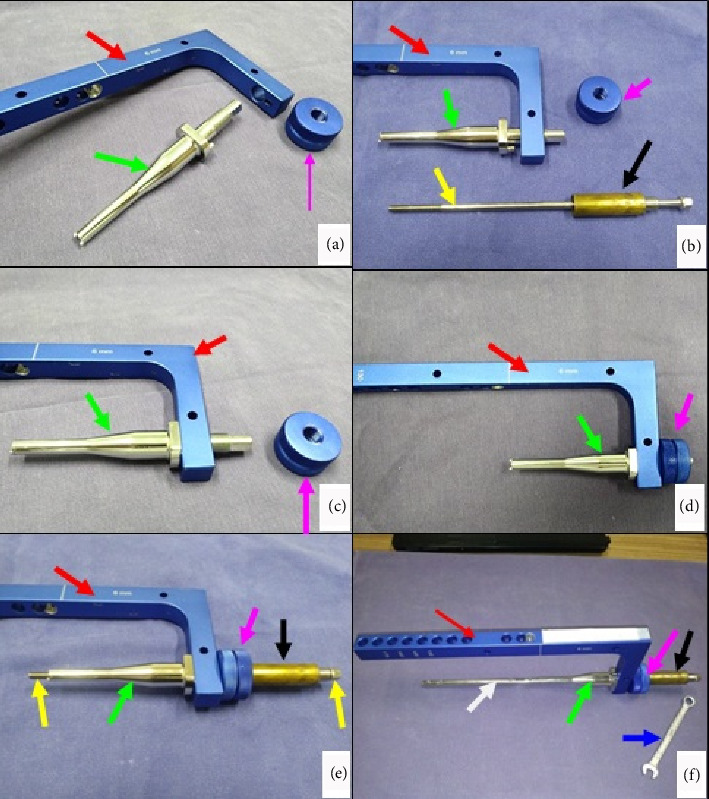
Photographic illustration of the external implantation guide (EIG) components associated with CD. (a) EIG (red arrow), nail connector (NC) (green arrow), and nail connector fixator (pink arrow). (b) EIG and CD with primary screw (yellow arrow) and spring retainer (black arrow). (c, d) The NC is inserted in the EIG and fixed with the nail connector fixator. (e) The primary screw of CD is inserted through the cannulated hole of NC. (f) The tightening wrench (blue arrow) is used to thread the screw inside the novel DCASIN.

The dynamic compression of the DCASIN is directly related to the CD. The other components of the system are derived from previous commercial models of interlocking nails. The CD consists of the following elements (Figure 1): a primary screw (PS) for compression, a compression measurer, a spring retainer housing a spring held by two washers, and a tightening wrench for the PS. The apparatus was designed to be inserted through the cannulated hole of the nail connector (NC) present in the external implantation guide (EIG) (Figure 2).

**Figure 3:**
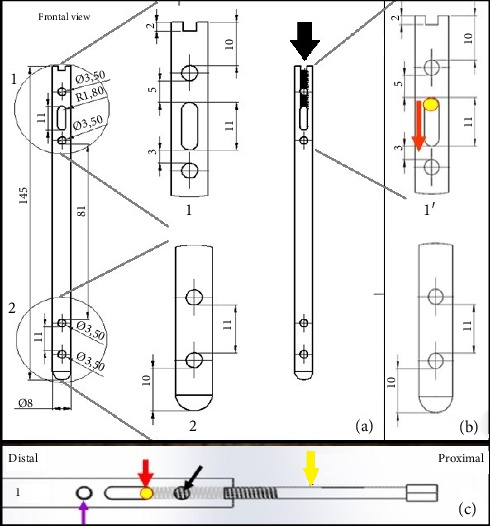
Prototype illustration of the body nail of the DCASIN, designed for dynamic compression when applied with the CD. (a, b) Frontal view with measurements in millimeters, depicting the oblong hole in the proximal portion of the nail (1), positioned between the two angle-stable holes. The distal portion (2) exhibits two angle-stable holes only. The threaded nail core (black arrow) originates from the proximal aspect of the nail and extends up to the oblong hole, facilitating the pushing of the implant (red arrow) inserted into the proximal aspect of the oblong hole. (c) The PS (yellow arrow) is inserted into the threaded nail core (black arrow) to make contact and push the Steinmann pin (red arrow), while the last hole of Portion 1 (purple arrow) is locked in an angle-stable position only after the PS is removed.

The body of the DCASIN was commissioned from a veterinary implant manufacturer (PROTOMED, São Paulo, Brazil), the same company responsible for producing the IC and the ASIN. The manufacture was performed using 316L steel, with an 8-mm diameter and a 145-mm length. It features holes designed for locking screws from the 3.5-mm system, comprising four angle-stable locking holes, with two proximal and two distal. The key design elements include an oblong hole in the proximal section, intended for dynamic compression, positioned between the two proximal angle-stable holes, and a threaded nail core up to the oblong hole.  Figure 3 shows the details of the design.

The authors hypothesized that upon applying the DC to the EIG, the PS would engage with the threaded nail core. Following the threading process, the nail would be secured, while the PS would displace distally within the nail core, pushing the Steinmann pin into the oblong hole. This action would lead to the distal displacement of the proximal bone fragment, resulting in compression between the two bone fragments. Importantly, this process would not result in any modification of the nail's position relative to the EIG, thereby ensuring the stability of the hole angles.

### 2.2. Compression Technique Execution

**Figure 4:**
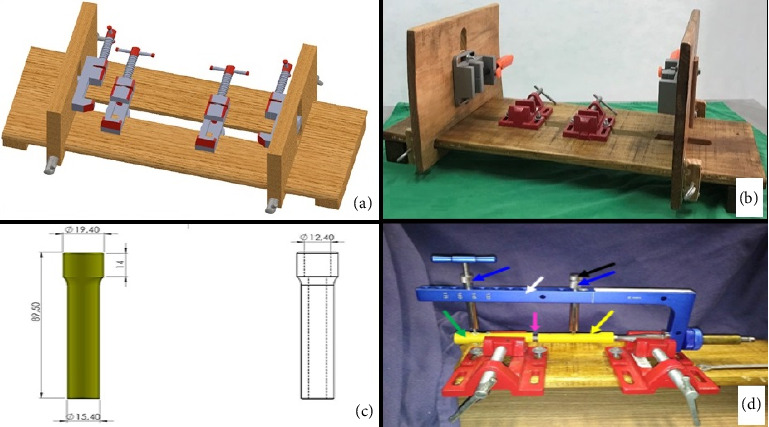
Prototype and photographic illustration for the modified test bench and synthetic specimens used for the assays for the dynamic compression of novel DCASIN and CD. (a) Prototype of the test bench, featuring two bench vises in the horizontal plane for specimen fixation and two others in the vertical plane for EIG fixation. (b) Test bench after the wood manufacturing. (c) Prototype of the specimens using PLA, with measures in millimeters and mimicking long cylindrical bones. (d) The final appearance of the modified test bench with the EIG, DCASIN, and CD. The distal specimen (green arrow) and the EIG (white arrow) were secured in the bench vises. The EIG was utilized with perforation guides (blue and black arrows) to insert angle-stable screws. An initial distance of 7 mm (pink arrow) was maintained between the proximal (yellow arrow) and distal specimens.

The technique execution and validation were conducted utilizing a modified test bench for interlocking nails (Figures 4(a) and 4(b)) [16], along with synthetic specimens designed to mimic cylindrical bones with transverse fractures [17, 18]. These specimens were three-dimensionally printed using polylactic acid (PLA) (Figures 4(c) and 4(d)) to match the density of cortical bones [19]. Additionally, the experimental setup included the EIG, DCASIN, and CD. The technique was replicated six times to ensure consistency, with the seventh execution performed using a specimen featuring a window in the region of the proximal holes (two angle-stable and one oblong hole) to illustrate the compression pathway.

**Figure 5:**
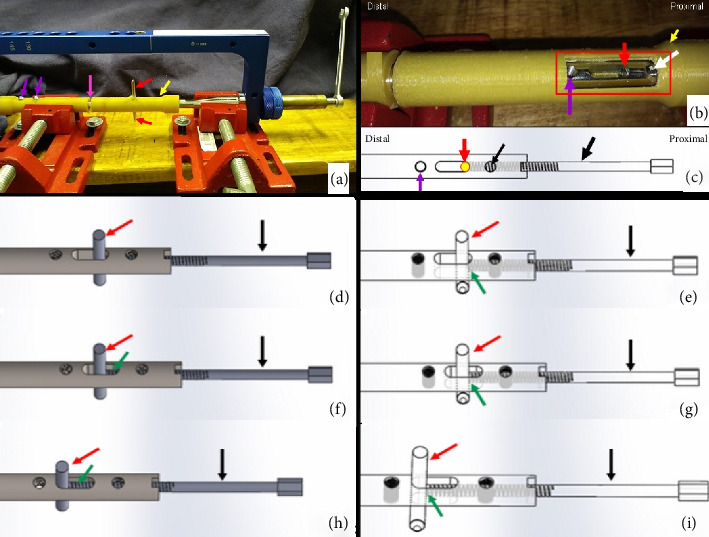
Sequential illustrative demonstration of the compression technique using the novel DCASIN and the CD. (a) The proximal specimen (yellow arrow) was free to axial motion, while the distal one was secured to the bench vise and fastened to the nail using two angle-stable screws (purple arrows). The PS was threaded along the nail core until it made contact with the Steinmann pin (red arrow), pushing it distally along the axial vector. This action led to the approximation of the distal specimen and resulted in compression of the initial gap (pink arrow). (b, c) The proximal specimen (yellow arrow) with a window to show the pathways. The red rectangle shows the site of implant insertion in the proximal position of the nail. The first (white arrow) and third (purple arrow) holes of the proximal portion were left unobstructed while the PS (black arrow) was inserted into the nail core. The sequence of figures in (d–i) demonstrates the anticipated displacement of the implant components during the execution of the compression technique. As the PS (black arrow) is threaded, the Steinmann pin (red arrow) is displaced distally into the oblong hole (green arrow).

The apparatus was constructed by inserting the nail into both specimens mimicking a transverse long bone fracture. The distal specimen and the EIG + DCASIN were secured in the bench vises, while the proximal specimen was free to axial motion (Figure 5). A distance of 7 mm was maintained between the two specimens.

The implantation of the DCASIN began with the insertion of two angle-stable locked screws in the distal portion using the EIG (Figure 5(a)). The axial dynamic compression was executed as follows:

1. The PS of the CD was inserted into the cannulated hole of the NC on the EIG, resulting in threading into the nail core;
2. On the proximal specimen, a 3.5-mm Steinmann pin was inserted into the proximal aspect of the nail's oblong hole through the EIG, passing through both cortical faces of the bone;
3. The PS of the CD was threaded using a wrench into the nail core, passing through the first proximal angle-stable hole of the nail and touching the Steinmann pin in the oblong hole (Figures 5(b) and 5(c));
4. During the insertion of the PS, the Steinmann pin was pushed in an axial and distal direction, resulting in the displacement of the proximal toward the distal end, while all other components of the device remained stable (Figure 5(d));
5. The proximal specimen's dislocation was performed until the compression was achieved against the distal specimen, and the axial movement of the proximal specimen could be measurable with the compression guide in the CD;
6. Subsequently, the second angle-stable hole of the proximal portion of the nail, immediately distal to the oblong hole, was fixed using a screw, ensuring the fixation of the proximal specimen in the compressed position;
7. The PS was unscrewed in order to allow the screw fixation into the first angle-stable hole in the proximal aspect of the nail;
8. After achieving the complete angle-stable fixation of the proximal fragment, the Steinmann pin was removed from the oblong hole.

The compression validation was based on the axial and distal displacement of the proximal specimen, effectively closing the initial 7-mm gap. Photographs were taken to document the proposed axial compression (Figure 5).

### 2.3. Objective Measurement of the Compression Technique and Comparative Analysis With CIN and ASIN

**Figure 6:**
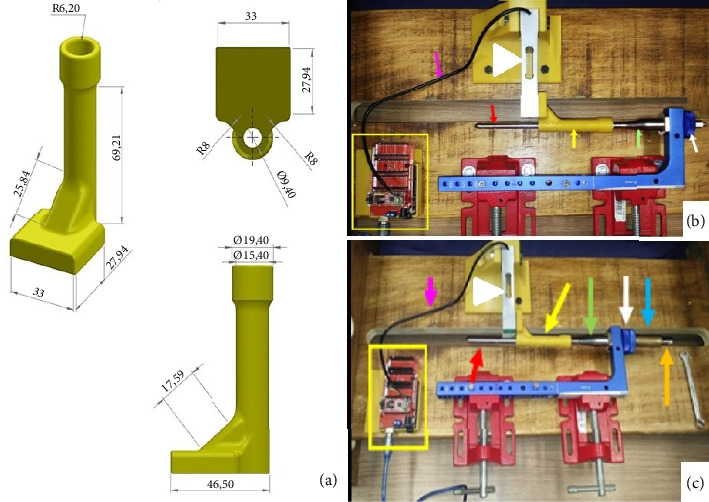
Prototype illustration and representation of the technique adopted for the objective measurement of the compression technique for the DCASIN compared to CIN and ASIN. (a) Description of specimens used for PLA three-dimensional printing, featuring millimeter measurements and cylindrical bone shapes, adapted with perpendicular terminals for intimate contact with the beam-type strain gauge load cell. (b) Test bench setup with the ASIN model (red arrow), showing the specimen (yellow arrow) in contact with the load cell (white arrowhead) and NC (green arrow) with the EIG (white arrow). The load cell (white arrowhead) was secured to the test bench using a sinew and connected to the Arduino/Genuino Uno (yellow rectangle) via a cable (pink arrow). (c) Test bench setup with the DCASIN, highlighting the CD (blue arrow) and PS (orange arrow).

Three different models of interlocking nails were used for this analysis. The first group (G1) consisted of CIN manufactured from 316L steel, the second group (G2) comprised the ASIN made from 316L steel, and the third group (G3) included the DCASIN. Each group was tested 10 times (10 tests per group, totaling 30 tests for this section), utilizing the same test bench described earlier. The test specimens were identical in material, measurements, and shape but were modified to have perpendicular terminals for intimate contact with the beam-type strain gauge load cell (Figure 6(a)).

The objective compression measurements were performed using a beam-type strain gauge load cell (Flintec, Betim-MG, Brazil, model SB61C-100 kg) [3] which was connected to the Arduino/Genuino Uno platform and the Arduino Integrated Development Environment (IDE) software (Version 1.8.8). This setup allowed for force measurements in grams (g). All devices were adapted to the test bench as described in Figure 6. All the sequence of the study was performed by a single surgeon.

For G1 and G2, the specimens were manually compressed against the load cell until a force of 400–500 g was achieved and stabilized for at least 60 s, simulating the manual compression that might be performed during real surgery. After this period and while maintaining the initial force, the screws (conventional or angle-stable) were inserted into the nail through the specimen. For G3, the specimens were initially kept at a 2-mm distance from the load cell, presuming that compression would be achieved using the CD. Therefore, the initial compression forces for G3 were 0 g. As the DCASIN (G3) was inserted into the specimen using the CD and PS (as described in Section 2.2), the compression forces were generated, and the specimen contacted the load cell. Compression performed until resistance from the PS was encountered during threading. The angle-stable screws of the DCASIN were inserted only after the PS was threaded.

Four moments were analyzed in the groups. For G1 and G2, the moments were as follows: prelocking (M1), just after the first screw insertion in the proximal nail (M2), after the second screw insertion in the proximal nail (M3), and 1 minute after the end of M3 (M4). For G3, the moments were as follows: just after the Steinmann pin insertion in the oblong hole (M1), after the compression of the specimen to the load cell (M2), after the insertion of the distal angle-stable screw in the proximal nail (M3), and 1 minute after the end of the procedure (M4), which included the removal of the PS and insertion of the first angle-stable screw in the proximal nail.

### 2.4. Finite Element Analysis (FEA)

The FEA was conducted using the SolidWorks 3D CAD software (Dassault Systèmes, SolidWorks Corporation, Waltham, MA, USA) to evaluate the stress generated during the DCASIN compression process, with a focus on the interaction between the proximal Steinmann pin and the proximal specimen. The analysis was performed in two stages under compression: (1) using a PLA bone specimen and (2) considering the natural properties of the bone tissue. The primary distinction between the two approaches was the tissue properties.

**Figure 7:**
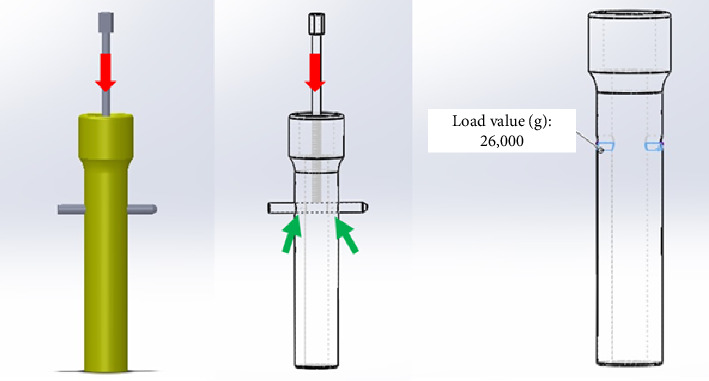
Graphical representation of finite element analysis execution, simulating the axial load exerted by the Steinmann pin on the bone specimen's orifice in both cortices. During the compression process, the primary screw applied axial pressure (red arrow), exerting force on the pin at two points on the distal surface of the cortical orifice (green arrow). A load of 26,000 g was applied, representing the maximum force value recorded during the compression test.

For Stage 1, the bone specimen design was identical to that used for 3D printing (as described in Section 2.2), incorporating the same dimensions and material (PLA), which were implemented in the software. The PLA properties adopted were a modulus of elasticity of 5 GPa, a Poisson's ratio of 0.3, and a compressive yield stress of 40 GPa. The finite element mesh was configured according to the default settings recommended by SolidWorks Simulation, employing a medium-density mesh. Each triangular element measured 2.322 mm by 0.116 mm and incorporated four Jacobian points. The distal portion of the proximal specimen was designated as the stable geometry and served as the simulated contact surface to mimic the compression technique. To simulate the axial force exerted by the pin on the proximal fragment during compression, the forces were applied to the specimen's orifice, including both cortical regions, on the distal surface of the orifice (Figure 7). The load used for the analysis was 26,000 g, which corresponds to the maximum force recorded during the compression test.

For Stage 2, the same process described previously was employed, except for the material properties input into the software. In this analysis, the mechanical properties of the bone specimen were adjusted from PLA values to those representatives of the natural adult cortical bone tissue from a large breed dog, specifically an elastic modulus of 17 GPa, a Poisson's ratio of 0.3, and a compressive yield stress of 0.115 GPa [20–22]. The mesh, fixed points, direction, magnitude, and points of load application remained consistent with the initial configuration.

### 2.5. Statistical Analysis

All statistical tests were performed using the SigmaPlot software Version 12.0. The statistical analysis was divided into two parts to compare the internal values within each group and the values between the groups, considering each evaluation moment. All values were subjected to the Shapiro–Wilk normality test. Those with non-normal distribution were subsequently tested using the nonparametric Friedman test, followed by Tukey's test. Groups with values that assumed a normal (parametric) distribution were tested using repeated measures analysis of variance (ANOVA) with Tukey's post hoc test.

In the second part, the groups were compared with each other, considering the values at each evaluation moment. Again, the Shapiro–Wilk normality test was applied. Values with a normal distribution were evaluated using one-way ANOVA, followed by Tukey's test for comparisons showing significant differences. For values with a non-normal distribution, the Kruskal–Wallis test was used, followed by Dunn's test. The significance level established for all tests was 5% (*p* ≤ 0.05).

## 3. Results

### 3.1. Compression Technique Execution

**Figure 8:**
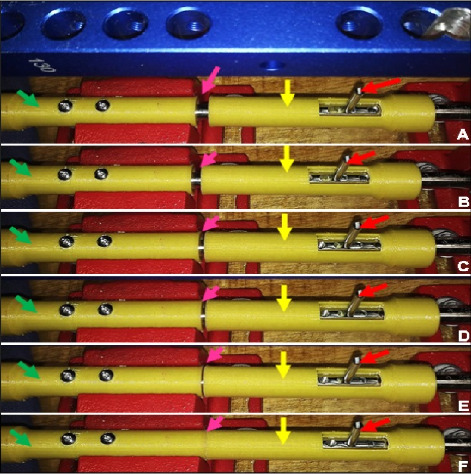
Sequential photographic representation of the interfragmentary compression achieved by the novel DCASIN. (a) Initially, the distal specimen (green arrow) was immobilized, while the proximal one (yellow arrow) remained free to axial motion. The positioning screw (PS) was advanced through the nail core to guide the Steinmann pin into the oblong hole (red arrow). (b–f) The sequential axial motion of the proximal specimen corresponding to the advancement of the PS. The distal displacement of the proximal specimen led to the complete closure of the initial 7-mm gap (pink arrow).

In this phase, six specimens containing transverse fractures were used to test the dynamic compression effect of the novel DCASIN. Initially, it was confirmed that the specimens used, printed in PLA, were suitable for mimicking the shape of long bones and that the test bench was sufficient to ensure the stability and assembly of the apparatus. All the specimens (100%) achieved complete fracture reduction and compression, as verified by macroscopic evaluation (Figure 8), maintaining the compressed position after the insertion of the two angle-stable screws in the proximal portion of the nail.

### 3.2. Objective Measurement of the Compression Technique and Comparative Analysis With CIN and ASIN

**Figure 9:**
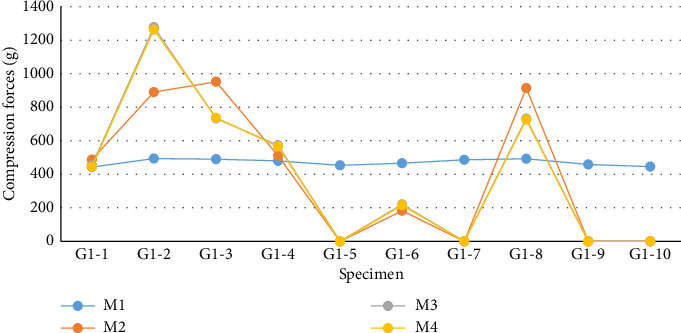
Graphical demonstrations of individual values corresponding to the compression force exerted by the CIN (G1) at different evaluation moments, captured by the Arduino/Genuino Uno computational platform, in the 10 specimens used.

In this phase of the experiment, 10 compression force measurements were conducted per group, totaling 30 measurements. For G1 (Figure 9 and Table 1), which utilized CIN, the values obtained from the tests exhibited a nonparametric distribution according to the Shapiro–Wilk normality test (*p* < 0.050). Therefore, the evaluation of G1 was based on the medians using the nonparametric Friedman test, revealing no statistical difference between the evaluation moments (*p*=0.903). Upon specific analysis of the values, it was noted that 50% (5/10) of the samples from G1 showed a decrease in compressive preload immediately at the transition from M1 to M2. In four of these five samples, the compression value dropped to 0 g at M2 and remained so until total nail blockage (M4). In specimens numbered 2, 3, and 8 (3/10), an increase in compressive load was observed during the tests. Only G1-2 showed a progressive increase during the moments, ending with the highest load observed in this group (1263 g).

**Figure 10:**
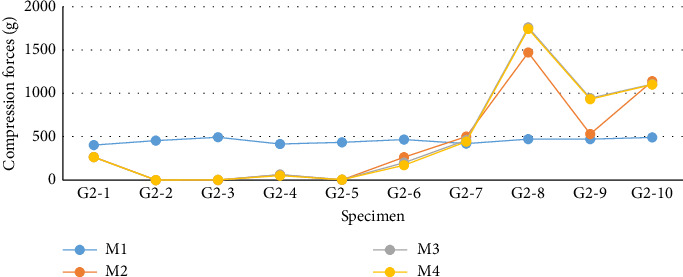
Graphical demonstrations of individual values corresponding to the compression force exerted by the ASIN (G2) at different evaluation moments, captured by the Arduino/Genuino Uno computational platform, in the 10 specimens used.

The values for G2 (Figure 10 and Table 2) exhibited a nonparametric distribution according to the Shapiro–Wilk normality test (*p* < 0.050). Therefore, median values were considered for conducting the nonparametric Friedman test. Despite differences between the moments, none of them proved to be statistically significant (*p*=0.422). In this group, 60% (6/10) of the samples showed a decrease in compressive preload at M2. Overall, the decreases were smaller than those observed in G1, with only two samples (G2-2 and G2-3) showing a measurement of zero (0 g) at the second evaluation moment (M2). An increase in compressive load was detected in 40% (4/10) of the samples, with three out of these four samples (G2-8, G2-9, and G2-10) showing a progressive increase at each evaluation moment. After complete blockage of the ASIN used in G2, Sample G2-8 exhibited the highest compressive load, reaching 1743 g.

**Figure 11:**
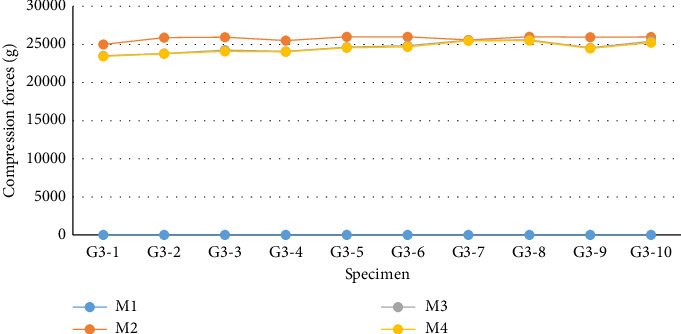
Graphical demonstrations of individual values corresponding to the compression force exerted by the DCASIN (G3) at different evaluation moments, captured by the Arduino/Genuino Uno computational platform, in the 10 specimens used.

Regarding the measurements in G3 (Figure 11 and Table 3), according to the Shapiro–Wilk normality test, the compressive loads followed a parametric distribution (*p*=0.611). For this reason, the mean values of the samples at each evaluation moment were considered for conducting repeated measures ANOVA, which showed a significant difference within the group (*p* < 0.001). Due to this significance, the evaluation moments of G3 were compared using Tukey's test. The means of M2, M3, and M4 were higher than the mean at M1 evaluation (*p* < 0.001), where the compressive load was 0 g. The average compressive load recorded at M2 was higher than the values recorded at M3 (*p* < 0.001) and M4 (*p* < 0.001). According to Tukey's test, the decrease observed from M3 to M4 was not statistically significant (*p*=0.947).

The sample values of G1, G2, and G3 were compared at each evaluation moment. At all moments, the values of the three groups exhibited a parametric distribution in the Shapiro–Wilk test; thus, the mean compressive loads recorded were considered for performing ANOVA followed by Tukey's post hoc test. In comparing the values recorded at the first evaluation moment (M1), there was a significant difference between G1 and G2 compared to G3 (*p* < 0.01), which was not subjected to preload. At the subsequent moments (M2, M3, and M4), G3 showed significantly higher compressive forces than those identified in G1 and G2 (*p* < 0.001), indicating greater compressive capacity in this group compared to the others. Regardless of the evaluation moment considered, there was no statistically significant difference between the loads recorded by G1 and G2. The fluctuations observed in these groups at different evaluation moments did not reach the minimum significance level of 5% (*p* ≤ 0.05).

### 3.3. FEA

**Figure 12:**
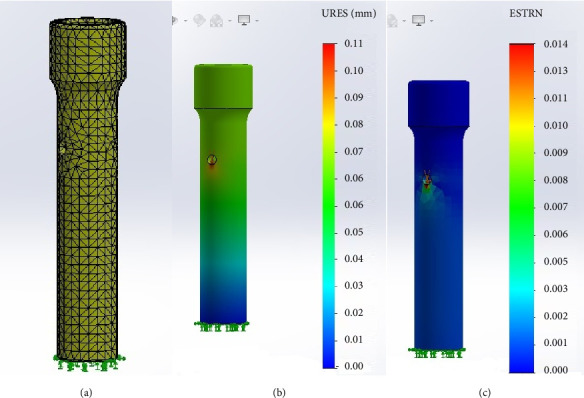
Finite element analysis results for the polylactic acid (PLA) specimen. (a) Definition of finite element mesh, with the distal portion of the specimen designated as the stable geometry, and green arrows representing the simulated contact surface, as observed during the compression technique. (b) Displacement under axial force of 26,000 g. (c) Equivalent deformation in the distal region of the orifice following the application of an axial force of 26,000 g.

Initially, the FEA was performed using the PLA specimen. The software generated the specimen's mesh (Figure 12(a)), and the resulting displacement and equivalent deformation were evaluated (Figures 12(b) and 12(c)). The application of a 26,000-g force resulted in minimal deformation of the specimen, with a maximum recorded displacement of 0.1 mm. This displacement was macroscopically negligible, aligning with the macroscopic assessment of the specimen during the compression test.

**Figure 13:**
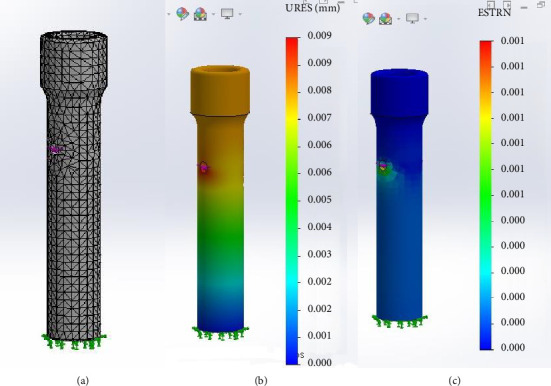
Finite element analysis results for the natural adult cortical bone tissue specimen. (a) Definition of finite element mesh, with the distal portion of the specimen designated as the stable geometry, and green arrows representing the simulated contact surface, as observed during the compression technique. (b) Displacement under axial force of 26,000 g. (c) Equivalent deformation in the distal region of the orifice following the application of an axial force of 26,000 g.

The second analysis utilized the natural adult cortical bone tissue as the specimen. The results under compression were similar to those obtained with the PLA specimen (Figure 13). The maximum compression force of 26,000 g did not cause any deformation or macroscopic dislocation in the cortical bone, indicating that its structural integrity remained intact.

## 4. Discussion

The intramedullary nail designed to generate interfragmentary compression associated with CD is unprecedented in veterinary orthopedics. Existing models in human orthopedics feature an oblong hole that allows axial movement and, consequently, interfragmentary compression [23–26]. However, although such models limit torsional instability, they do not provide angle-stable locking. For this reason, it is believed that if similar models were used in veterinary orthopedics, they would likely yield unsatisfactory results, as non–angle-stable models have been found inadequate for veterinary use due to the gap between the nail and the locking implant, resulting in inadequate bone stabilization [5, 6, 13, 27, 28]. Therefore, the new DCASIN model promoted interfragmentary compression and reduced instability with angle-stable locking, while the compression technique did not induce bone deformation.

One of the complications reported in the literature during the execution of the intramedullary nail implantation technique is the incorrect insertion of the locking implant, with this error being more frequently observed in the most distal hole of the nail [1, 29, 30]. This issue may be further exacerbated in compressive models due to bone fragment displacement [11, 12], an occurrence not observed in the proposed model here, given the maintenance of distal fragment staticity together with the EIG for DCASIN implantation. On one hand, the mechanism developed for compression in the DCASIN model elaborated here allows for full stability between the external locking guide and the nail, maintaining the concentric configuration of the drill holes and subsequent screw insertions, proving effective in all tests conducted here. On the other hand, with equal importance, this technique was not inhibitive to achieve satisfactory compression, as evidenced by the 7-mm displacement in all specimens, as well as the progressively increasing compressive forces significantly higher than those of commercially available nail models.

The failure of the implant in the oblong hole is one of the most reported complications in compressive nail models in humans [25, 31, 32]. This occurs because, once inside the oblong hole, the locking implant remains perpendicular to axial forces and is therefore subjected to bending during both intraoperative and postoperative interfragmentary compression [26]. Therefore, it is preferable to apply to these nail implants' devoid of threads, as the thread flanks reduce the implant's moment of inertia, making it less resistant to bending loads [26]. For the conceptualization of the technique and apparatus proposed in this research, even though there was no external resistance to the specimens caused by adjacent soft tissues and body mass, as in clinical tests, it was decided to block the oblong hole with a 3.5-mm Steinmann pin. Ultimately, it was observed that there was no failure of the Steinmann pin in any of the samples during compression, a fact that, combined with the aforementioned arguments, encourages the use of this implant in future clinical testing modalities in this novel DCASIN. Additionally, the pin can be removed once the other locks in the proximal fragment are secured, unlike compressive nail models in humans [25].

For nail models lacking a compression mechanism, it is described that manual techniques performed during nail implantation may generate compression [33, 34]. Considering this information, in the comparative tests of interfragmentary compressive load, G1 and G2 were subjected to manual preload, standardized within the range of 400–500 g. It was observed that the load was not maintained, and the values of G1 and G2 were heterogeneous, with many samples showing zero compressive load (0 g). These values are consistent with the literature reports that indeed nails lacking a compression system are unable to generate and/or maintain compressive loads [25]. In comparison to G3, the values of G1 and G2 were lower at all evaluation moments (*p* < 0.001), except for the first evaluation moment, where G3 did not show any load as it was intentionally not subjected to preload for verification purposes. The ideal compressive load to promote primary consolidation is not well defined, but it is described that high loads, exceeding 3000 N (300 kg), can impair fracture healing [24, 35]. Previous authors [25] demonstrated, through biomechanical evaluation, that the compressive load generated by available compressive nail models in medicine can range from 700 to 1400 N (70–140 kg), with this load directly related to the torque applied to the CD. The loads applied in this research were lower than those mentioned above; however, they approached the compressive values recorded by LCP plates, which reach about 30 kg when a screw is implanted in the dynamic compression mode [3].

In biomechanical tests to evaluate compressive loads generated with interlocking nails, it is necessary for the load cell not to interfere with nail implantation or the specimen. At the same time, the specimen should maintain the largest possible contact surface with the load cell [25, 26]. In the methodology proposed here, the load cell was positioned laterally and perpendicular to the specimens, which were adapted with perpendicular terminals to the medullary canal to increase the contact surface with the load cell. This externally created area around the canal was efficient in deforming the load cell according to the compressive force applied, generating compressive load data without hindering the implantation technique. Furthermore, in line with previous studies [3, 25], it was observed that the load cell was effective in measuring the compressive force for the orthopedic implant proposed here.

Considering the greater forces exerted by the DCASIN during compression, it is essential to assess the potential for bone deformation [21, 22]. In this study, the FEA was conducted to evaluate the effects of compression forces, exerted by the pin, on both cortical regions. The results indicated that even when the maximum recorded force was applied, no significant damage was observed in either the PLA or cortical bone, confirming the safety of the compression technique. The adoption of 3D-printed PLA specimens in the present study, although it may be considered a limitation of the proposed evaluation technique, offers superior quality compared to the use of cadaveric bones for testing orthopedic implants [36, 37]. This approach ensures standardization of force resistance for all models used, generating more reliable results and requiring a smaller number of samples [36]. Additionally, the choice of the PLA material was based on its elastic modulus similar to that of the cortical bone tissue [19], which is essential to ensure that the specimen closely resembles the material the new implant model will be intended for [36, 37]. In addition, the FEA results indicated that the behavior of the bone samples was comparable to that of the cortical bone tissue, as previously indicated [19], ensuring more reliable outcomes and confirming that the proposed DCASIN did not induce deformation during the compression technique. Furthermore, the specimen shape for studies involving interlocking nails should be cylindrical to adequately mimic the intramedullary canal and allow for device implantation [13, 18, 26]. For this reason, and considering this as a preliminary study, all PLA specimens used in the tests mimicked a long bone with a transverse fracture and a medullary canal suitable for the implantation of an 8-mm diameter locked intramedullary nail. Despite the superior quality of PLA [36, 37] and the demonstrated viability of synthetic cylindrical specimens designed to simulate bones with transverse fractures [17, 18], future evaluations should utilize more realistic materials that closely mimic the density of both hard and soft tissues to ensure more accurate trials [38].

Based on the methodology used and the results obtained, it is acknowledged that the commercially available intramedullary nail models used in this research, both conventional and angle-stable (G1 and G2, respectively), were neither able to maintain compressive preload consistently nor were they able to promote additional compressive load under these methodological conditions. Therefore, these implants are incapable of promoting interfragmentary compression. In contrast, the novel DCASIN model associated with the CD was able to generate interfragmentary compression, achieving complete apposition of fragments separated by 7 mm without the risk of causing bone damage, as indicated by the compression forces obtained in this study. These findings support its reliability and safety as a viable option for long bone fracture fixation. Furthermore, it was possible to implant the DCASIN associated with the CD using the standard instruments described for commercially available interlocking nail models. Subsequently, further studies with *in vivo* biomechanical tests and clinical trials [7] are suggested to emphasize the effectiveness and usability of the new materials listed here, as well as to elucidate the clinical outcomes that their use could generate.

## 5. Conclusion

It was concluded that the design of the CD, combined with the novel DCASIN model, is sufficiently effective in promoting compression among the analyzed specimens and can generate and maintain high compressive loads without bone damage, unlike the other two nail models tested.

## Figures and Tables

**Table 1:** General group values corresponding to G1 related to the compression force at different evaluation moments.

	M1 (g)	M2 (g)	M3 (g)	M4 (g)
Median	473	335.5	337	330.5
Mean	471	393.9	399	395.3
Standard deviation	±19.95	±410.63	±434.30	±430.48

**Table 2:** General group values corresponding to G2 related to the compression force at different evaluation moments.

	M1 (g)	M2 (g)	M3 (g)	M4 (g)
Median	461	264.5	232	216.5
Mean	452.2	423	480.2	470.6
Standard deviation	±32.11	±510.52	±599.37	±596.35

**Table 3:** General group values corresponding to G3 related to the compression force at different evaluation moments.

	M1 (g)	M2 (g)	M3 (g)	M4 (g)
Median	0	25,954	24,595	24,525
Mean	0	25789.1	24626.7	24528.3
Standard deviation	±0	±330.33	±732.59	±711.61

## Data Availability

The data that support the findings of this study are available from the corresponding author upon reasonable request.

## Nomenclature

DCASIN: Dynamic compression angle-stable interlocking intramedullary nail
CD: Compression device
PS: Primary screw
ASIN: Angle-stable interlocking intramedullary nail
CIN: Conventional intramedullary nails
NC: Nail connector
EIG: External implantation guide
PLA: Polylactic acid
